# Interstitial lung diseases after hematopoietic stem cell transplantation: New pattern of lung chronic graft-versus-host disease?

**DOI:** 10.1038/s41409-022-01859-4

**Published:** 2022-10-29

**Authors:** Gabrielle Archer, Ingrid Berger, Louise Bondeelle, Constance de Margerie-Mellon, Stéphane Cassonnet, Régis Peffault de Latour, David Michonneau, Sylvie Chevret, Anne Bergeron

**Affiliations:** 1 Sorbonne Université, Paris, France; 2 Université Paris Cité, AP-HP, Hôpital Saint-Louis, Service de Pneumologie, F-75010 Paris, France; 3 Université Paris Cité, AP-HP, Hôpital Saint-Louis, Service de Radiologie, F-75010 Paris, France; 4 AP-HP, Hôpital Saint-Louis, Service de Biostatistique et Information médicale, F-75010 Paris, France; 5 Université Paris Cité, AP-HP, Hématologie-Greffe, Hôpital Saint-Louis, Paris, France; 6 Université Paris Cité, UMR 1153 CRESS, ECSTRRA Team, F-75010 Paris, France; 7 Hôpitaux Universitaires de Genève, Service de Pneumologie, Genève, Switzerland

**Keywords:** Haematological diseases, Risk factors

## Abstract

Bronchiolitis obliterans syndrome (BOS) after allogeneic HSCT is the only formally recognized manifestation of lung chronic graft-versus-host disease (GVHD). Other lung complications were reported, including interstitial lung diseases (ILDs). Whether ILDs belong to the spectrum of lung cGVHD remains unknown. We compared characteristics and specific risk factors for both ILD and BOS. Data collected from consecutive patients diagnosed with ILD or BOS from 1981–2019 were analyzed. The strength of the association between patient characteristics and ILD occurrence was measured via odds ratios estimated from univariable logistic models. Multivariable models allowed us to handle potential confounding variables. Overall survival (OS) was estimated using the Kaplan-Meier method. 238 patients were included: 79 with ILD and 159 with BOS. At diagnosis, FEV1 was lower in patients with BOS compared to patients with ILD, while DLCO was lower in ILD. 84% of ILD patients received systemic corticosteroids, leading to improved CT scans and pulmonary function, whereas most BOS patients were treated by inhaled corticosteroids, with lung-function stabilization. In the multivariable analysis, prior thoracic irradiation and absence of prior treatment with prednisone were associated with ILD. OS was similar, even if hematological relapse was more frequent in the ILD group. Both complications occurred mainly in patients with GVHD history.

## Introduction

Allogeneic hematopoietic stem cell transplantation (HSCT) is a curative treatment for several hematological malignancies as well as immune deficiencies and hemoglobinopathies [1]. Despite the major progress made since the emergence of HSCT, specific complications can occur and impair the prognosis of patients [2]. Among them, chronic graft-versus-host disease (GVHD) is a leading cause of death and impaired quality of life [3, 4]. Chronic GVHD resembles a connective tissue disease with multiple organ involvement [5]. The National Institute of Health in its latest consensus conference suggests criteria for diagnosing and scoring the severity of chronic GVHD. “Diagnostic signs” described for each organ are considered sufficient for the clinician to make the diagnosis of chronic GVHD. The pulmonary manifestations of chronic GVHD are considered highly morbid forms by the 2020 consensus of the NIH due to their associated morbidity and mortality [6, 7]. Bronchiolitis obliterans syndrome (BOS) is the only formally recognized manifestation of lung chronic GVHD and refers to a functional respiratory profile, namely, a new-onset obstructive lung disease (OLD) with air trapping. The most common histological counterpart of BOS is obliterative bronchiolitis. In a prospective cohort, we found a cumulative incidence of BOS at 3 years after HSCT of 10% [8]. We also identified early risk factors for BOS, which were the use of peripheral blood stem cells (PBSCs), the occurrence of a lower respiratory tract infection before day 100, and a 10% decline in the forced expiratory volume in 1 second (FEV1) between the allograft and day 100 [8]. Many other risk factors have been reported in retrospective studies. In addition to BOS, other noninfectious late-onset pulmonary complications after HSCT (LONIPCs) have been described [9], including diffuse interstitial lung disease (ILD), whose 3-year cumulative incidence was found to be 5% [8, 10]. Post-HSCT ILD has been described more recently than BOS and it was associated with restrictive lung disease (RLD). Initially identified only as organizing pneumonia [11], ILDs have been shown by more-recent studies to be possibly due to other histological patterns [12–15]. In a retrospective histological study, we found that post-HSCT ILDs may correspond to several histological patterns, such as organizing pneumonia (OP) and nonspecific interstitial pneumonia (NSIP), predominantly, as well as diffuse alveolar damage, lymphocytic interstitial pneumonia, and pleuroparenchymal fibroelastosis (PPFE) [16]. Strikingly, in almost all cases, interstitial pathology coexisted with histological lesions of obliterative bronchiolitis. This association was found in other studies of postallogeneic HSCT PPFE or NSIP in which, when histological data were available, lesions of obliterative bronchiolitis were present in 70–100% of cases [14, 17]. It was previously suggested that the chronic inflammation and fibrosis characteristic of both OLD and RLD were associated with GVHD and the intensity of HCT conditioning [18]. Indeed, the few studies dedicated to post-HSCT overall ILD or, more specifically, to post-HSCT-OP found a strong association with acute and/or chronic GVHD [10, 12, 13, 19, 20]. Conditioning regimens, including total body irradiation and cyclophosphamide, were also associated with post-HSCT-OP [11]. Although ILD is not currently consensually considered a manifestation of chronic pulmonary GVHD, both the frequent histologic association with obliterative bronchiolitis and clinical association with other manifestations of GVHD raise the question of whether ILDs could be part of a spectrum of lung cGVHD.

The goal of our study was to compare the characteristics and outcomes of patients who developed ILD after allogeneic HSCT with those who developed BOS. We used a retrospective observational cohort of 238 consecutive patients from the diagnosis of BOS or ILD, managed in the respiratory department of the Saint Louis Hospital, Paris, France.

## Patients and methods

At our site, all allogeneic HSCT recipients routinely underwent pulmonary function tests (PFTs) before transplant, at day 100 and at 6, 12, 18, 24, and 36 months post-HSCT. PFT was performed using a body plethysmograph (Jaeger Masterscreen Body; Jaeger; GmBH; Wurburg, Germany). The diffusing capacity of carbon monoxide (DLCO) was measured using the single-breath method, and the results were adjusted to the last available hemoglobin level. When PFT was abnormal and/or when respiratory symptoms occurred, patients underwent chest computed tomography (CT) and a respiratory infectious work-up. In the case of an abnormal CT scan and if the clinical condition allowed it, patients underwent bronchoscopy and bronchoalveolar lavage (BAL) with an extensive search for viruses, bacteria and fungi. The total and differential cell counts in the BAL fluid were also analyzed. If the CT scan was normal, nasal swabs were performed to test for viruses, and sputum was collected for both bacterial and fungal analyses.

All consecutive allogeneic HSCT recipients diagnosed with BOS or ILD in our department between 1981 and 2019 were included in the study. Data were collected from individual medical records. Clinical data and PFT, BAL, nasal-swab and sputum findings were collected. The diagnosis and severity of acute and chronic GVHD were reported based on clinical grading scores [5, 21].

BOS was defined as previously described [22, 23]: (1) absence of respiratory infection at the time of PFT and (2) either a new-onset FEV1 < 75% of predicted or a decline of >10% in FEV1 from the pretransplant value and (3) either FEV1/vital capacity (VC) < 0.7 or a concomitant decrease in both FEV1 and VC < 80% of predicted, with a total lung capacity (TLC) > 80% of the predicted value [22, 23], and absence of infiltrative opacities on chest imaging.

ILD was diagnosed as previously described [12], i.e., when infiltrative opacities were present on HRCT, and (1) no pathogen was identified in the respiratory samples (BAL and/or nasal aspirate and/or sputum); (2) no clinical or radiological improvement was observed despite broad antimicrobial treatment; and/or (3) no pathogen was found on the lung biopsy (if available). For patients who had a lung biopsy, we classified the pathologic patterns as previously described [16]. While the diagnosis of ILD is not dependent on PFTs, PFTs are obtained in the majority of affected patients, assist in differentiating BOS from ILD, contribute to severity assessment of lung dysfunction, have prognostic value during follow-up of patients with ILD and are included in the description of these patients.

CT scans performed before HSCT, at the time of ILD diagnosis, and during follow-up were reviewed by an experienced radiologist (CDM) and three pulmonologists (LB, IB, GA). The following features were recorded: ground glass opacities, consolidation, signs of fibrosis (reticulation, septal lines, honeycombing, bronchiectasis), and emphysema. To assess lesion distribution, each lung was divided into three areas (upper/medial/lower) from the lung apices to the domes of the diaphragm. The extent of the scanographic lesions was graded semiquantitatively as less than 5%, 5–20%, 20–50%, and more than 50%. Conclusions were reached by consensus. Pretransplant HRCT and any scans performed between the time of HSCT and the diagnosis of ILD were also reviewed to ensure the absence of a previous ILD.

This retrospective study was approved by the institutional review board of the French Learned Society for Respiratory Medicine (CEPRO 2020-063) and the data collected was anonymized prior to processing (Redcap software).

### Statistical analysis

Summary statistics (i.e., median, interquartile range, and percentages) are reported. Comparison of baseline groups used the nonparametric Wilcoxon rank sum test and the exact Fisher test. As this study focused on post-HSCT ILD, patients who developed both BOS and ILD were included in the ILD group. Second, we compared the patient characteristics according to the occurrence of either BOS, ILD, or both.

Given only patients with BOS or ILD were selected, no incidence of those events could be computed in such a case-control design regarding the assessment of risk factors. Thus, the strength of the association between patient characteristics and the occurrence of ILD was measured by odds ratios (ORs) estimated from univariable logistic models. Multivariable models allowed us to handle potential confounding variables, including prognostic factors selected on univariable analyses based on a *p*-value below 0.10.

The design also included a follow-up of patients after BOS or ILD, providing a cohort study after diagnosis. Overall survival (OS) calculated from the date of ILD or BOS diagnosis until the date of death from any cause was estimated using the Kaplan-Meier method, with the log-rank test used for comparison purposes across baseline groups. Cumulative incidence of relapse was estimated in a competing risk setting, where deaths free of relapse competed with relapses. The prognostic value of ILD compared to BOS was measured on each outcome by the hazard ratio (HR) estimated from univariable and then multivariable Cox models in a similar way as described above, to adjust on prognostic factors.

Missing values for predictors were imputed by the mode if less than 10%. *P*-values less than 0.05 were considered statistically significant. All statistical analyses were performed using R 4.0.3 (R Foundation for Statistical Computing).

## Results

A total of 238 consecutive patients who were diagnosed with ILD (*n* = 79, 33%) or BOS (*n* = 159, 67%) after allogeneic HSCT between November 1981 and December 2019 were included in the study (the flow chart is available in the supplementary material, e-Figure 1). Distribution of diagnoses over time is shown in the supplementary material, e-Figure 2. Forty patients with ILD were previously reported [12]. Patient characteristics at the time of HSCT are summarized in Table 1. Male sex was more frequently associated with BOS than with ILD (52% males with BOS vs. 23% males with ILD; *p* < 0.0001). HLA-matched 9/10 donors were more frequently observed in the BOS than in the ILD group (20% vs. 5%), with significantly more female donor/male recipient mismatches among BOS patients (*p* = 0.034). No difference in conditioning regimens was observed between the BOS and ILD groups, including the consideration of treatments known to be associated with drug-related ILDs, namely, busulfan-based regimens, cyclophosphamide-based regimens, or total body irradiation. 207 patients had a pre-transplant HRCT available, including 154 patients with BOS and 53 patients with ILD. Among them, emphysema was present for 15 patients (10 patients with BOS and 5 patients with ILD) before HSCT; no other abnormality was detected on pre-transplant CT-scan in BOS patients. Among the 53 patients with ILD, two had ground glass opacities<5% and one had fibrosis <5%. No further abnormalities were found on pre-transplant lung CT scan.

**Table 1:** Patient characteristics at the time of allogeneic HSCT, according to the presence of BOS or ILD.

	BOS	ILD	*p*-value
	*N* = 159	*N* = 79	
Age at transplant (years)	43 [27–57]	46.6 [32–56]	0.32
Male	83 (52%)	18 (23%)	<0.0001
History of smoking	66 (42%)	42 (53%)	0.098
Underlying disease			
AL	79 (50%)	30 (38%)	0.098
Lymphoma	26 (16%)	16 (20%)	0.47
Myeloma	5 (3%)	6 (8%)	0.19
Myelodysplastic syndrome	17 (11%)	13 (16%)	0.22
Chronic myeloid leukemia	13 (8%)	4 (5%)	0.44
Other	19 (12%)	10 (13%)	1.00
Prior HSCT			
Autologous	22 (14%)	10 (13%)	0.37
Allogeneic	6 (4%)	3 (4%)	1
Prior thoracic irradiation	4 (3%)	6 (8%)	0.087
Status of disease at transplant			
1st complete response	66 (42%)	34 (43%)	0.90
2nd complete response	32 (20%)	14 (18%)	
Other	59 (38%)	31 (39%)	
Missing data	2	0	
Stem cell source			
PBSCs	135 (85%)	67 (85%)	0.11
Bone marrow	20 (13%)	6 (7.5%)	
Cord blood	4 (2%)	6 (7.5%)	
Donor HLA status			
Geno-identical donor	76 (48%)	34 (43%)	0.49
Haplo-identical donor	8 (5%)	5 (6%)	0.76
Unrelated donor	75 (47%)	40 (51%)	0.68
HLA-match 10/10	58 (78%)	32 (80%)	0.028
HLA-match 9/10	15 (20%)	2 (5%)	
Others	1 (1%)	3 (7.5%)	
Missing data	1 (1%)	3 (7.5%)	
Donor sex			
Female	63 (43%)	32 (53%)	0.22
Matched donor-recipient sex			
Female donor to male recipient	37 (26%)	6 (10%)	0.034
Male donor to female recipient	46 (32%)	21 (35%)	
Sex match	62 (43%)	33 (55%)	
Missing data	14	19	
Conditioning regimen			
Myeloablative	78 (49%)	40 (50%)	0.78
Nonmyeloablative	81 (51%)	39 (50%)	
Busulfan-based	76 (48%)	38 (48%)	1.00
Cyclophosphamide-based	54 (34%)	27 (34%)	1.00
Total body irradiation	37 (23%)	28 (35%)	0.06
Anti-thymocyte globulin	50 (31%)	29 (37%)	0.47
GVHD prophylaxis			
Cyclosporine/methotrexate	48 (30%)	18 (23%)	0.29
Cyclosporine/mycophenolate mofetil	86 (54%)	41 (52%)	0.86
Posttransplant cyclophosphamide	2 (1%)	3 (4%)	0.42

*BOS* bronchiolitis obliterans syndrome, *ILD* interstitial lung disease, *AL* acute leukemia, *HSCT* hematopoietic stem cell transplantation, *PBSC* peripheral blood stem cells *HLA* human leukocyte antigen, *GVHD* graft-versus-host disease

### Patient characteristics at the time of ILD/BOS diagnosis

The median time to diagnosis from HSCT was 13 months [IQR 7–24] in the BOS group and 15 months [IQR 7–26] in the ILD group. The characteristics of the patients at the time of ILD or BOS are shown in Table 2. Although a vast majority of patients in both groups had a history of GVHD (acute GVHD: ILD *n* = 44, 56%, BOS *n* = 118, 76%; cGVHD: ILD *n* = 60, 75%, BOS *n* = 148, 92%), patients with BOS had significantly more acute and chronic GVHD than ILD patients. Consistently, patients with ILD were less likely to receive immunosuppressive treatment for cGVHD, including prednisone, than patients with BOS before and at the time of lung disease diagnosis.

**Table 2:** Patient characteristics at the time of BOS or ILD diagnosis.

	BOS	ILD	*p*-value
Patients	*N* = 159	*N* = 79	
Year of Diagnosis	2013, July	2016, June	0.016
≥2013	85 (53%)	52 (66%)	0.07
Acute GVHD	118 (76%)	44 (56%)	0.003
Grading of aGVHD			0.035
0	38 (25%)	35 (44%)	
1	24 (16%)	12 (15%)	
2	57 (38%)	22 (28%)	
3	29 (19%)	10 (13%)	
4	3 (2%)	0 (0)	
Missing data	8	0	
Chronic GVHD before or at diagnosis of BOS or ILD	148 (92%)	60 (75%)	0.0003
Maximum severity of chronic GVHD			0.90
Mild	27 (18%)	10 (17%)	
Moderate	64 (43%)	28 (47%)	
Severe	57 (39%)	22 (37%)	
Immunosuppressive treatment for cGVHD before and at diagnosis of BOS or ILD			
None	10 (6%)	27 (34%)	<0.0001
Prednisone for cGVHD	137 (86%)	49 (62%)	<0.0001
Prednisone* in cGVHD patients	141 (89%)	54 (68%)	0.00016
Mycophenolate-mofetil	28 (18%)	11 (14%)	0.58
Methotrexate	3 (2%)	1 (1%)	0.58
Ciclosporin	28 (18%)	18 (23%)	0.38
mTOR inhibitor	11 (7%)	3 (4%)	0.4
Azathioprine	5 (3%)	1 (1%)	0.67
Ruxolitinib	14 (9%)	2 (2%)	0.098
Anti-TNF	3 (2%)	2 (2%)	1
Other	40 (25%)	14 (18%)	0.25
Ongoing IS treatment at the time of diagnosis of BOS/ILD			
Prednisone	78 (49%)	30 (38%)	0.13
Cyclosporine	56 (35%)	27 (34%)	1.00
Mycophenolate mofetil	18 (11%)	9 (11%)	1.00

*BOS* bronchiolitis obliterans syndrome, *ILD* interstitial lung disease, *GVHD* graft-versus-host disease, *IS* immunosuppressive

*Whatever the indication of prednisone (either BOS, ILD, or cGvHD)

### Characteristics of ILD and BOS

Symptoms at the diagnosis of ILD or BOS and treatments initiated for lung disease are summarized in Table 3. Of note, among patients with ILD, 28 (35%) required oxygen supply at ILD diagnosis and 19 (24%) were admitted in intensive care unit.

**Table 3:** Symptoms at the time of BOS/ILD diagnosis and treatment of BOS/ILD.

	BOS *N* = 159	ILD *N* = 79	*P*-value
Symptoms at diagnosis			
Cough	84 (53%)	54 (68%)	0.0258
Dyspnea	82 (52%)	66 (84%)	<0.0001
No symptoms	60 (38%)	30 (38%)	1.00
Oxygen supply at diagnosis	19 (13%)	28 (35%)	<0.0001
ICU admission at diagnosis	0 (0%)	19 (24%)	<0.0001
Treatment of ILD/BOS*			
Macrolides	83 (52%)	12 (15%)	<0.0001
LABA/Inhaled steroids	156 (99%)	17 (22%)	<0.0001
Systemic steroids	78 (49%)	66 (84%)	<0.0001

*BOS* bronchiolitis obliterans syndrome, *ILD* interstitial lung disease, *ICU* intensive care unit, *LABA* long-acting beta(2) agonist

*Treatment given specifically for ILD/BOS at diagnosis

In current practice, the diagnosis and characterization as well as the follow-up of ILD relies mostly on CT scans and PFT, whereas the diagnosis and follow-up of BOS is mainly based on PFT. Evidence of air trapping by expiratory CT scan is one of the supporting features of BOS according to the NIH [5]; however, its specificity is limited [8]. Therefore, we focused on the description of the CT scan of the ILD group. The CT scan pattern mostly consisted of peribronchovascular alone or both peribronchovascular and subpleural consolidations (81%) and ground glass opacities (87%) with no cranio-caudal predominance. Twenty-nine (37%) patients had a CT scan pattern compatible with OP, and 10 (13%) had pleuroparenchymal fibroelastosis. CT scan analysis at the time of diagnosis and first revaluation are shown in Table 4. Seventy-one percent of bronchiectasis cases were found at diagnosis, 25% of which regressed completely at the first re-evaluation. Overall, the CT scan abnormalities evolved positively (46% consolidation, 74% ground glass opacity at reevaluation). Twelve (15%) patients had a normal CT scan at reevaluation. Notably, lung histology was available for 6 patients, of which one case showed NSIP, one case showed diffuse alveolar damage, one case showed OP, one case showed an association of NSIP and OP, and 2 cases were inconclusive.

**Table 4:** Lung CT scan findings in patients at ILD diagnosis and at first reassessment.

	At diagnosis, *N* = 79				At reassessment, *N* = 65			
	Extension (%)				Extension (%)			
Imaging findings	<5	5–20	20–50	>50	<5	5–20	20–50	>50
Consolidation (%)	27 (34)	33 (42)	4 (5)	0	22 (34)	8 (12)	0	0
Ground glass opacities (%)	23 (29)	31 (39)	11 (14)	4 (5)	16 (25)	22 (34)	8 (12)	2 (3)
Fibrosis (%)	42 (53)	14 (18)	3 (4)	0	19 (29)	16 (25)	1 (1)	0
Reticulation (%)	10 (13)				6 (9)			
Septal lines (%)	4 (5)				2 (3)			
Traction bronchiectasis (%)	56 (71)				36 (55)			
Honeycombing (%)	0				0			
Distribution								
Subpleural (%)	15 (19)				9 (14)			
Peri-broncho-vascular (%)	26 (33)				16 (25)			
Mixed (%)	38 (48)				26 (40)			
Predominance								
Superior (%)	28 (35)				16 (25)			
Middle (%)	20 (25)				12 (18)			
Inferior (%)	17 (22)				14(21)			
Pattern								
OP (%)	29 (37)							
PPFE (%)	10 (13)							
Undetermined (%)	40 (50)							

*OP* organizing pneumonia, *PPFE* pleuroparenchymal fibroelastosis

Table 5 reports the PFT findings at the time of diagnosis of BOS/ILD. Patients with BOS showed lower FEV1. DLCO was lower at diagnosis in the ILD group (43% versus 57.5%) and then was comparable during the follow-up. Spaghetti plots of the FEV1 trajectory before and after the diagnosis of ILD and BOS are shown in e-Figure 3.

**Table 5:** PFT characteristics at the time of BOS or ILD diagnosis and thereafter.

	BOS	ILD	*p*-value
	*N* = 145	*N* = 69	
At the time of diagnosis			
FEV1 %predicted	63.5 [46;74]	70 [51.25;87.5]	0.022
FVC %predicted	75 [64.75;86]	71 [54.75;87.25]	0.20
DLCOc %predicted	57.5 [49;67.5]	43 [32;57]	0.002
Last measurement			
Follow-up, months	41.3 [11.4;85.9]	25.9 [8.2;56.9]	0.063
FEV1 %predicted	64 [45.5;81.5]	79 [51;101]	0.013
FVC %predicted	79 [65.5;92]	82 [58;100]	0.66

*PFTs* pulmonary function tests, *BOS* Bronchiolitis obliterans syndrome, *ILD* interstitial lung disease, *FEV1* forced expiratory volume in 1 s; *FVC* forced vital capacity, *DLCO* diffusing capacity of carbon monoxide

### Predictive factors for ILD

Table 6 summarizes the predictive factors for the occurrence of ILD, as selected from univariable analyses. Prior thoracic irradiation and the absence of immunosuppressive treatment at the time of diagnosis were associated with an increased occurrence of ILD.

**Table 6:** Predictive factors for the development of ILD rather than BOS after allogeneic HSCT: Results of the univariable and multivariable logistic regression analyses.

	Univariable model		Multivariable model	
	OR (95% CI)	*p*-value	OR (95% CI)	*p*-value
Period of diagnosis				
>2013	1.68 (0.96 to 2.93)	0.07	1.06 (0.88–1.27)	0.55
Female	3.70 (2.01–6.82)	<0.0001	1.25 (0.93–1.68)	0.14
History of smoking	1.11 (0.98–1.25)	0.088	1.13 (0.94–1.35)	0.20
AL	0.62 (0.36–1.08)	0.089	0.96 (0.80–1.29)	0.71
Sex match				
Match	1.00		1.00	
Donor F—Recipient M	0.81 (0.69–0.96)	0.012	0.88 (0.69–2.53)	0.29
Donor M—Recipient F	0.97 (0.84–1.11)	0.64	0.87 (0.66–1.15)	0.34
Prior thoracic irradiation	3.18 (0.87–11.6)	0.08	1.80 (1.29–2.53)	0.0011
HLA match				
other	1.00		1.00	
10/10	0.67 (0.42–1.07)	0.095	0.55 (0.24–1.30)	0.18
aGVHD	0.4 (0.23–0.72)	0.002	1.08 (0.86–1.37)	0.50
cGVHD	1.00 (0.58–1.71)	1.00	0.78 (0.34–1.78)	0.56
Prednisone (treatment for cGVHD)	0.26 (0.14–0.5)	<0.0001	0.65 (0.50–0.85)	0.002

*OR* odds ratio, *CI* confidence interval, *AL* acute leukemia, *F* female, *M* male, aGVHD acute graft-versus-host disease, *cGVHD* chronic graft-versus-host disease, *IS* immunosuppressive, *ILD* interstitial lung disease.

### Outcomes according to ILD versus BOS

The median follow-up after ILD was 41 months [IQR: 17–80] and 50 months [IQR: 21–98] after BOS. Twenty-one (27%) patients had a recurrence of ILD when the corticosteroid therapy was decreased or stopped.

**Fig. 1:**
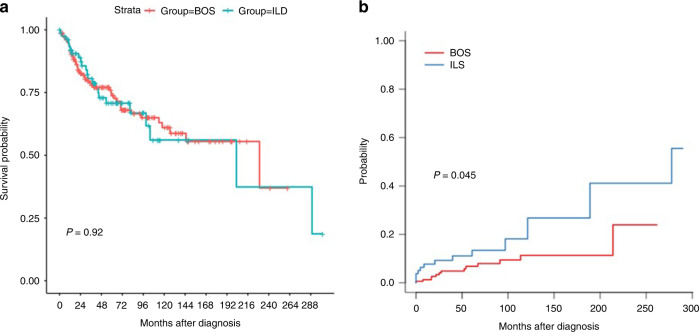
Outcomes according to the type of diagnosis (BOS vs. ILD). (**a**) Overall survival; (**b**) Cumulative incidence of hematological relapse.

Observed OS was similar between groups, with an observed survival at 36 months of 78.7% (95%CI, 72.3 to 85.7) for BOS and 80.6% (95%CI, 71.6 to 90.8) for ILD, and at 5 years of 73.8% (95%CI, 66.6 to 81.8) and 70.8% (95%CI, 60.0 to 83.5), respectively (Fig. 1). Unadjusted hazard of death in the ILD group was 1.03 (95%CI, 0.62 to 1.70); it was unchanged (HR = 1.04, 95%CI, 0.58 to 1.86; *p* = 0.88) after adjusting on age, sex, tobacco history, acute leukemia, prior thoracic irradiation or GvHD; it became 1.00 (95%CI, 0.56 to 1.77) when further adjusting on period of diagnosis.

Causes of death differed between groups. Seven patients (33%) in the ILD group died from respiratory failure versus 8 patients (18%) in the BOS group, whereas 18 patients (41%) died from infection in the BOS group versus 4 patients (19%) in the ILD group.

The cumulative incidence of hematological relapse was increased in the ILD group, with 29 relapses versus 54 in the BOS group (*p* = 0.045, Fig. 1)This increase was erased after adjusting for potential confounders, namely, sex, history of thoracic irradiation, and acute GVHD (HR = 2; 95% CI 0.81–4.98; *p* = 0.13).

### Association of BOS and ILD

Notably, 18 patients developed both complications simultaneously or sequentially based on both the CT scan and the PFT patterns. Patient characteristics are summarized in e-Table 1 and e-Table 2 (see the supplementary Appendix). Overall, patients developing ILD and BOS showed striking similarities with the BOS population, i.e., a higher proportion of patients developing either acute or chronic GVHD with lower FEV1.

## Discussion

This large retrospective study allowed us to compare the characteristics of postallogeneic HSCT ILD to BOS. We found similarities, such as a similar median time from HSCT, up to 85% of patients who had received peripheral blood stem cells as a stem cell source in both groups of patients, a high proportion of patients who developed GVHD, although this was more striking for patients with BOS who also developed more severe acute GVHD. We also found discrepancies between the two groups, with the BOS group showing a higher proportion of males, HLA mismatch, and sex disparities between donor and recipient; patients with ILD received less steroids for GVHD from HSCT than BOS patients, thoracic irradiation prior to HSCT was found to be associated with the development of post-HSCT-ILD. The treatment given for the lung dysfunction differed between the BOS and the ILD group, with a majority of patients treated with inhaled corticosteroids in the BOS group and with systemic corticosteroids in the ILD group. Although OS did not differ between groups, the causes of death varied, with more cases of respiratory failure in the ILD group and more cases of infection in the BOS group.

ILD is now a recognized post-HSCT pulmonary complication [7]. However, it is unclear whether ILD is part of the spectrum of chronic pulmonary GVHD. Our study reinforces this hypothesis. Indeed, in addition to the previous demonstration of concomitant histological lesions of ILD and BOS in the lungs of allogeneic HSCT recipients [16], the clinical association with chronic GVHD in 70–87% of cases has already been described in smaller series [11, 13, 24]. Furthermore, some of our patients developed a mixed clinical and functional phenotype associating both BOS and ILD. A number of factors associated with ILD in other settings are known, such as environmental, drug-related or genetic factors. Allogeneic HSCT recipients are administered many drugs that can induce ILD. We did not find an association between the type of conditioning regimen, including total body irradiation, and the occurrence of an ILD. However, we found a strong association between pretransplant thoracic irradiation and ILD. Although based on small sample of patients, this finding is consistent with the results of our prospective study focused on early risk factors for LONIPCs [8]. It could be postulated that lung injury secondary to this irradiation could promote the subsequent occurrence of ILD in a similar way to the phenomenon described for the occurrence of OP after irradiation for breast cancer [25].

Some pathophysiological mechanisms underlying the development of obliterative bronchiolitis, namely, peribronchiolar infiltration by T lymphocytes (CD4+) and antigen-presenting cells, the presence of alloantibodies, and the role of pathogenic Th17 cells, share similarities with chronic GVHD in other organs [26, 27]. Conversely, the pathophysiological characteristics of post-HSCT ILDs are not known. However, a certain number of elements argue for an immunological process: the occurrence of an ILD in patients receiving little or no immunosuppressive treatment and the efficacy of prednisone on thoracic imaging and respiratory function.

The survival of patients who developed BOS or ILD was similar, being approximately 70% at 5 years after diagnosis of ILD or BOS. However, the cause of death differed between groups, with a higher rate of respiratory failure in the ILD group. In contrast to patients with BOS, patients with ILD were less at risk of dying from infection. Notably, patients with BOS had received more steroids between HSCT and BOS diagnosis than patients who developed ILD while specific treatment of ILD included more steroids than treatment of BOS.

Interestingly, the hematological relapse rate was higher among patients with ILDs. It could be postulated that the administration of systemic steroids for the treatment of ILD and not for BOS promoted these relapses. Although our results rely on a large cohort of patients, our study has intrinsic bias due to its retrospective and unicentric design. First, post-HSCT ILDs are heterogeneous and have different patterns, probably associated with different mechanisms and prognoses, as we previously showed for PPFE [28]. The small number of patients with each type of ILD did not allow them to be analyzed separately. However, this concern is similar for BOS, for which different phenotypes are being identified [7]. We had previously shown that the histologies of posttransplant ILDs were various [16]. Few of the patients in this study had a lung biopsy, which would have helped clarify their lung disease.

However, this is in line with the practices of most other adult HSCT centers [29]; indeed, on one hand, recent advances in the noninvasive diagnosis of respiratory infections often make it possible to rule out an infectious cause without a lung biopsy being required; on the other hand, although video-assisted lung biopsies are less invasive than open lung biopsies, they are still associated with morbidity and mortality; thus, lung biopsy is decreasingly indicated in these fragile patients, which reinforces the need to better phenotype them radioclinically according to pneumological standards which make it possible to differentiate BOS from ILDs on both PFT profile and CT scan pattern. Nevertheless, whether lung histology could quantify the proportion of pulmonary inflammation and fibrosis and guide treatment is currently questionable.

Finally, while ILDs in other contexts, on the one hand, and chronic GVHD, on the other hand, are frequently associated with the presence of circulating autoantibodies, these data were not available for our patients. We have previously described posttransplant ILDs associated with a specific clinical-biological picture of different connective tissue disorders [30]. Unfortunately, we also did not have available a precise clinical description of the patients in the current study.

In summary, ILDs must now be included in the spectrum of postallogeneic HSCT pulmonary complications. They mainly occur in patients with chronic GVHD. Formally integrating ILD into the spectrum of chronic pulmonary GVHD would require a better understanding of its pathophysiology. Although we have shown specificities between BOS and ILD, the poor prognosis is the same.

## Supplementary information

supplementary

## Data Availability

The complete dataset can be requested from the author.
