# Supplementary material for: Interstitial lung diseases after hematopoietic stem cell transplantation: New pattern of lung chronic graft-versus-host disease?
Source: Bone Marrow Transplant. 2022 Oct 29;58(1):87–93. doi: 10.1038/s41409-022-01859-4 (PMC9812763; doi:10.1038/s41409-022-01859-4)

**Supplementary material**

Interstitial lung diseases after hematopoietic stem cell transplantation: New pattern of lung chronic graft-versus-host disease?

Gabrielle Archer, Ingrid Berger, Louise Bondeelle, Constance de Margerie-Mellon, Stéphane Cassonnet, Régis Peffault de Latour, David Michonneau, Sylvie Chevret, Anne Bergeron

**e-Table 1**: Patient characteristics at the time of allogeneic HSCT (supplementary appendix)

|  | |  | BOS | ILD | BOS and ILD | p-value |
| --- | --- | --- | --- | --- | --- | --- |
|  | | | N=159 | N=61 | N=18 |  |
| Age at transplant (years) | | | 43 [27–57] | 47 [34–59] | 38 [25–52] | 0.21 |
| Male | | | 83 (52%) | 12 (20%) | 6 (33%) | <0.0001 |
| History of smoking | | | 66 (42%) | 30 (50%) | 12 (67%) | 0.21 |
| Underlying disease | | |  |  |  |  |
|  | | AL | 79 (50%) | 25 (41%) | 5 (28%) | 0.15 |
|  | | Lymphoma | 26 (16%) | 12 (20%) | 4 (22%) | 0.64 |
|  | | Myeloma | 5 (3%) | 4 (7%) | 2 (11%) | 0.14 |
|  | | Myelodysplastic syndrome | 17 (11%) | 7 (11%) | 6 (33%) | 0.036 |
|  | | Chronic myeloid leukemia | 13 (8%) | 3 (5%) | 1 (6%) | 0.84 |
|  | Others | | 19 (12%) | 10 (16%) | 0 | 0.0001 |
| Prior HSCT | | |  |  |  |  |
|  | | Autologous | 22 (14%) | 6 (10%) | 4 (22%) | 0.28 |
|  | | Allogeneic | 6 (4%) | 2 (3%) | 1 (6%) | 0.73 |
| Prior thoracic irradiation | | | 4 (3%) | 3 (5%) | 3 (17%) | 0.028 |
| Status of disease at transplant | | |  |  |  |  |
|  | | 1st complete response | 66 (42%) | 25 (41%) | 9 (50%) | 0.93 |
|  | | 2nd complete response | 32 (20%) | 12 (20%) | 2 (11%) |  |
|  | | Other | 59 (38%) | 24 (39%) | 7 (39%) |  |
|  | | Missing data | 2 | 0 | 0 |  |
| Stem cell source | | |  |  |  |  |
|  | | PBSCs | 135 (85%) | 51 (84%) | 16 (89%) | 0.13 |
|  | | Bone marrow | 20 (12.5%) | 4 (6%) | 2 (11%) |  |
|  | | Cord blood | 4 (2.5%) | 6 (10%) | 0 |  |
| Donor HLA status | | |  |  |  |  |
|  | | Geno-identical donor | 76 (48%) | 27 (44%) | 7 (39%) | 0.74 |
|  | | Haplo-identical donor | 8 (5%) | 4 (7%) | 1 (6%) | 0.90 |
|  | | Unrelated donor | 75 (47%) | 30 (49%) | 10 (56%) | 0.81 |
|  | | HLA-match 10/10 | 58 (78%) | 22 (81%) | 10 (100%) | 0.065 |
|  | | HLA-match 9/10 | 15 (20%) | 2 (7%) | 0 |  |
|  | | Other | 1 (1%) | 3 (11%) | 0 |  |
| Donor sex | | |  |  |  |  |
|  | | Female | 63 (43%) | 22 (52%) | 10 (56%) | 0.43 |
| Matched donor-recipient sex | | Female donor to male recipient | 37 (25%) | 2 (5%) | 4 (22%) | 0.033 |
|  | | Male donor to female recipient | 46 (32%) | 15 (36%) | 6 (33%) |  |
|  | | Sex match | 62 (42%) | 25 (59%) | 8 (44%) |  |
|  | | Missing data | 14 | 19 | 0 |  |
| Conditioning regimen | | |  |  |  |  |
|  | | Nonmyeloablative | 81 (52%) | 31 (51%) | 8 (44%) | 0.85 |
|  | | Busulfan-based | 76 (48%) | 28 (46%) | 10 (56%) | 0.79 |
|  | | Cyclophosphamide-based | 54 (34%) | 22 (36%) | 5 (28%) | 0.84 |
|  | | Total body irradiation | 37 (24%) | 23 (38%) | 5 (28%) | 0.11 |
|  | | Antithymocyte globulin | 50 (32%) | 23 (38%) | 6 (33%) | 0.68 |
| GVHD prophylaxis | | |  |  |  |  |
|  | | Cyclosporine/methotrexate | 48 (30%) | 13 (21%) | 5 (28%) | 0.42 |
|  | | Cyclosporine/mycophenolate mofetil | 86 (54.1) | 32 (52%) | 9 (50%) | 0.86 |
|  | | Post-transplant cyclophosphamide | 2 (1%) | 3 (5%) | 0 | 0.19 |

BOS: bronchiolitis obliterans syndrome; ILD: interstitial lung disease; AL: acute leukemia; HSCT: hematopoietic stem cell transplantation; PBSC: peripheral blood stem cells; GVHD: graft-versus-host disease

**e-Table 2**: Patient characteristics at the time of BOS and/or ILD diagnosis (supplementary appendix)

|  |  | | | | | | BOS | ILD | BOS and ILD | p-value |
| --- | --- | --- | --- | --- | --- | --- | --- | --- | --- | --- |
| Patients | | | | | | | N=159 | N=61 | N=18 |  |
| Time from HSCT (months) | | | | | | | 13 [7–24] | 13.4 [6.7–22.6] | 23.2 [7.4–43.7] | 0.29 |
| Acute GVHD | | | | | | | 118 (76%) | 30 (49%) | 14 (78%) | 0.0005 |
| Grading of aGVHD | | | | | | |  |  |  |  |
|  | 0 | | | | | | 38 (25%) | 31 (51%) | 4 (22%) | 0.04 |
|  | 1 | | | | | | 24 (16%) | 8 (13%) | 4 (22%) |  |
|  | 2 | | | | | | 57 (38%) | 16 (26%) | 6 (33%) |  |
|  | 3 | | | | | | 29 (19%) | 6 (10%) | 4 (22%) |  |
|  | 4 | | | | | | 3 (2%) | 0 | 0 |  |
|  | NA | | | | | | 8 | 0 | 0 |  |
| Chronic GVHD | | | | | | | 148 (92%) | 42 (69%) | 17 (94%) |  |
| Maximum severity of chronic GVHD | | | | | | |  |  |  |  |
|  | Mild | | | | | | 27 (18%) | 8 (14%) | 1 (6%) | < 0.0001 |
|  | Moderate | | | | | | 64 (43%) | 23 (39%) | 5 (28%) |  |
|  | Severe | | | | | | 57 (39%) | 11 (19%) | 11 (61%) |  |
| Immunosuppressive treatment for cGVHD | | | | | | |  |  |  |  |
|  | None | | | | | | 10 (6%) | 25 (41%) | 2 (11%) | < 0.0001 |
|  | Prednisone | | | | | | 137 (86%) | 33 (54%) | 16 (89%) | < 0.0001 |
|  | Mycophenolate-mofetil | | | | | | 28 (18%) | 8 (13%) | 3 (17%) | 0.78 |
|  | Methotrexate | | | | | | 3 (2%) | 1 (2%) | 0 | 1 |
|  | Ciclosporin | | | | | | 28 (18%) | 10 (16%) | 8 (44%) | 0.032 |
|  | mTOR inhibitor | | | | | | 11 (7%) | 2 (3%) | 1 (6%) | 0.67 |
|  | Azathioprine | | | | | | 5 (3%) | 1 (2%) | 0 | 1 |
|  | Ruxolitinib | | | | | | 14 (9%) | 2 (3%) | 0 | 0.28 |
|  | Anti-TNF | | | | | | 3 (2%) | 1 (2%) | 1 (6%) | 0.42 |
|  | Other | | | | | | 40 (25%) | 9 (15%) | 5 (28%) | 0.22 |
| Ongoing IS treatment at the time of diagnosis of BOS/ILD | | | | | | |  |  |  |  |
|  | Prednisone | | | | | | 78 (49%) | 21 (34%) | 9 (50%) | 0.13 |
|  | Cyclosporine | | | | | | 56 (35%) | 18 (30%) | 9 (50%) | 0.29 |
|  | Mycophenolate mofetil | | | | | | 18 (11%) | 5 (8%) | 4 (22%) | 0.23 |
| CT scan | | | | | | |  |  |  |  |
|  | | | Extension (%) | | | |  |  |  |  |
| Imaging findings | | | <5 | 5–20 | 20–50 | >50 |  |  |  |  |
|  | Consolidation | |  |  |  |  |  |  |  |  |
|  | GGO | |  |  |  |  |  |  |  |  |
|  | Fibrosis | |  |  |  |  |  |  |  |  |
|  |  | Reticulation |  | | | | 0 | 7 (11%) | 3 (17%) | < 0.0001 |
|  |  | Septal lines |  | | | | 0 | 2 (3%) | 2 (11%) | 0.004 |
|  |  | Bronchiectasis |  | | | | 0 | 47 (77%) | 9 (50%) | < 0.0001 |
|  |  | Honeycombing |  | | | | 0 | 0 | 0 |  |
| Distribution | | | | | | |  |  |  |  |
|  | Subpleural | |  | | | | 0 | 9 (14%) | 6 (33%) | 0.028 |
|  | Peri-broncho-vascular | |  | | | | 0 | 18 (30%) | 8 (44%) |  |
|  | Mixed | |  | | | | 0 | 34 (56%) | 4 (22%) |  |
| Predominance | | | | | | |  |  |  |  |
|  | Superior | |  | | | | 0 | 23 (38%) | 5 (28%) | < 0.0001 |
|  | Middle | |  | | | | 0 | 16 (26%) | 4 (22%) | < 0.0001 |
|  | Inferior | |  | | | | 0 | 0 | 1 (6%) | 0.076 |
| PFT | | | | | | |  |  |  |  |
|  | FEV1 %predicted | | | | | | 63.5 [46; 74] | 76.5 [55; 92] | 55 [40–62] | 0.0008 |
|  | FVC %predicted | | | | | | 75 [64.75; 86] | 74 [56.25; 90] | 61 [51; 81] | 0.12 |
|  | DLCOc %predicted | | | | | | 57.5 [49; 67.5] | 43 [32; 57] | 54 [40; 69] | 0.005 |

|  |  | | BOS | ILD | BOS and ILD | p-value |
| --- | --- | --- | --- | --- | --- | --- |
| Patients | | | N=159 | N=61 | N=18 |  |
| Acute GVHD | | | 118 (76%) | 30 (49%) | 14 (78%) | 0.0005 |
| Grading of aGVHD | | |  |  |  |  |
|  | 0 | | 38 (25%) | 31 (51%) | 4 (22%) | 0.04 |
|  | 1 | | 24 (16%) | 8 (13%) | 4 (22%) |  |
|  | 2 | | 57 (38%) | 16 (26%) | 6 (33%) |  |
|  | 3 | | 29 (19%) | 6 (10%) | 4 (22%) |  |
|  | 4 | | 3 (2%) | 0 | 0 |  |
|  | NA | | 8 | 0 | 0 |  |
| Chronic GVHD | | | 146 (92%) |  |  |  |
| Maximum severity of chronic GVHD | | |  |  |  |  |
|  | Mild | | 27 (18%) | 8 (14%) | 1 (6%) | < 0.0001 |
|  | Moderate | | 64 (43%) | 23 (39%) | 5 (28%) |  |
|  | Severe | | 57 (39%) | 11 (19%) | 11 (61%) |  |
| Immunosuppressive treatment for cGVHD | | |  |  |  |  |
|  | None | | 10 (6%) | 25 (41%) | 2 (11%) | < 0.0001 |
|  | Prednisone | | 137 (86%) | 33 (54%) | 16 (89%) | < 0.0001 |
|  | Mycophenolate-mofetil | | 28 (18%) | 8 (13%) | 3 (17%) | 0.78 |
|  | Methotrexate | | 3 (2%) | 1 (2%) | 0 | 1.00 |
|  | Ciclosporin | | 28 (18%) | 10 (16%) | 8 (44%) | 0.032 |
|  | mTOR inhibitor | | 11 (7%) | 2 (3%) | 1 (6%) | 0.67 |
|  | Azathioprine | | 5 (3%) | 1 (2%) | 0 | 1.00 |
|  | Ruxolitinib | | 14 (9%) | 2 (3%) | 0 | 0.28 |
|  | Anti-TNF | | 3 (2%) | 1 (2%) | 1 (6%) | 0.42 |
|  | Other | | 40 (25%) | 9 (15%) | 5 (28%) | 0.22 |
| Ongoing IS treatment at the time of diagnosis of BOS/ILD | | | 108 (68%) |  |  |  |
|  | Prednisone | | 78 (49%) | 21 (34%) | 9 (50%) | 0.13 |
|  | Cyclosporine | | 56 (35%) | 18 (30%) | 9 (50%) | 0.29 |
|  | Mycophenolate mofetil | | 18 (11%) | 5 (8%) | 4 (22%) | 0.23 |
| CT scan | | |  |  |  |  |
| Reticulation | | | 0 | 7 (11%) | 3 (17%) | <0.0001 |
| Septal lines | | | 0 | 2 (3%) | 2 (11%) | 0.004 |
| Bronchiectasis | | | 0 | 47 (77%) | 9 (50%) | <0.0001 |
| Honeycombing | | | 0 | 0 |  |  |
| Distribution | | |  |  |  |  |
|  | Subpleural |  | 0 | 9 (14%) | 6 (33%) | 0.028 |
|  | Peri-broncho-vascular |  | 0 | 18 (30%) | 8 (44%) |  |
|  | Mixed |  | 0 | 34 (56%) | 4 (22%) |  |
| Predominance | | |  |  |  |  |
|  | Superior |  | 0 | 23 (38%) | 5 (28%) | <0.0001 |
|  | Middle |  | 0 | 16 (26%) | 4 (22%) | <0.0001 |
|  | Inferior |  | 0 | 0 | 1 (6%) | 0.076 |
| PFT | | |  |  |  |  |
|  | FEV1 %predicted | | 63.5 [46; 74] | 76.5 [55;92] | 55 [39.75;62] | 0.0008 |
|  | FVC %predicted | | 75 [64.75; 86] | 74 [56.25;90.25] | 61 [51;80.75] | 0.12 |
|  | DLCOc %predicted | | 57.5 [49; 67.5] | 43 [32;57] | 54 [39.75;68.75] | 0.005 |

BOS: bronchiolitis obliterans syndrome; ILD: interstitial lung disease; aGVHD: acute graft-versus-host disease; cGVHD: chronic graft-versus-host disease; IS: immunosuppressive; GGO: ground glass opacities; PFT: pulmonary function test; FEV1: forced expiratory volume in 1 second; FVC: forced vital capacity; DLCO: diffusing capacity of carbon monoxide

**e-Figure 1:** Flowchart of patients included in the analysis



**e-Figure 2:** Distribution of late onset noninfectious pulmonary complications over time


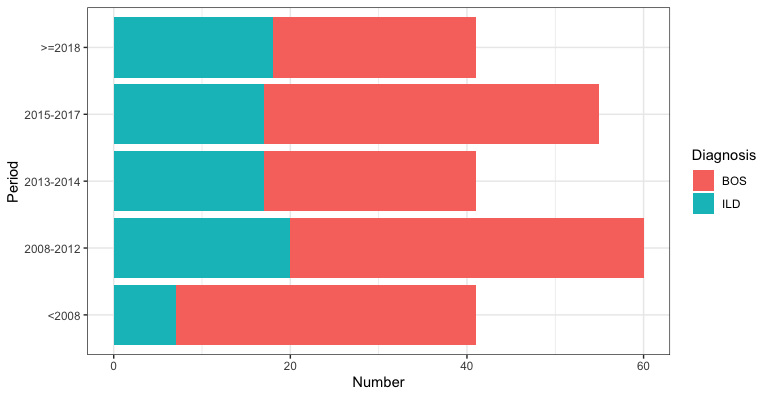


**e-Figure 3**: Spaghetti plot of the FEV1 trajectory according to the presence of ILD (A) vs. BOS (B). Time 0 refers to the time of diagnosis.

**A**


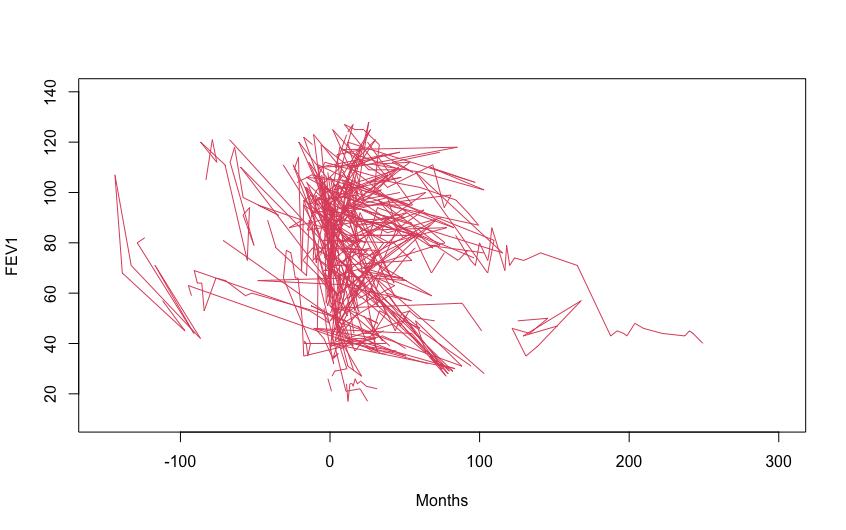


**B**

**
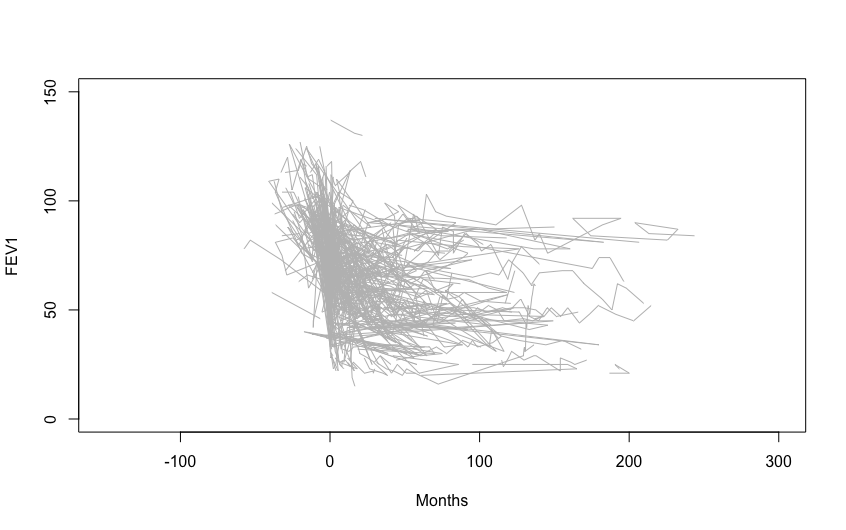
**

**e-Figure 4:** Overall survival of late onset noninfectious pulmonary complications according to time of diagnosis


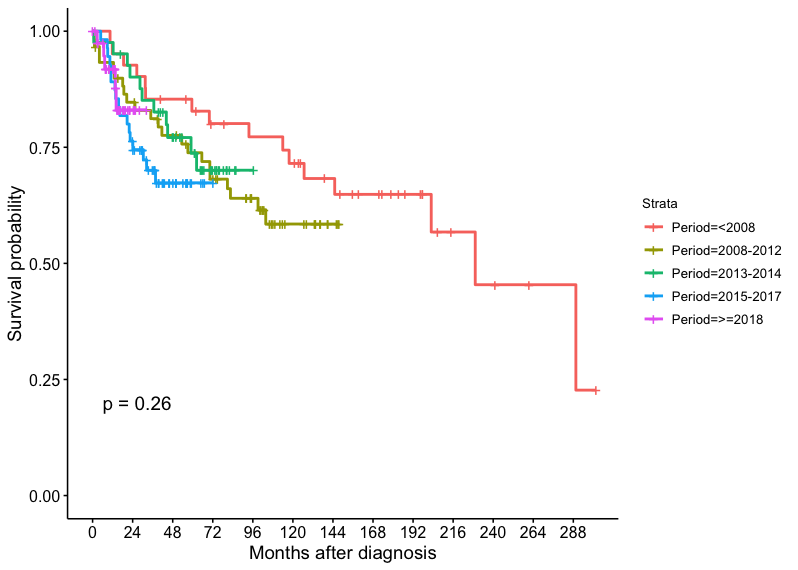

Supplement: Supplementary file 1 — supplementary [file 41409_2022_1859_MOESM1_ESM.docx]
